# Effect of *Watermelon Silver Mottle Virus* on the Life History and Feeding Preference of *Thrips palmi*

**DOI:** 10.1371/journal.pone.0102021

**Published:** 2014-07-10

**Authors:** Wei-Te Chen, Chien-Hao Tseng, Chi-Wei Tsai

**Affiliations:** Department of Entomology, National Taiwan University, Taipei, Taiwan; Volcani Center, Israel

## Abstract

Thrips-borne tospoviruses cause numerous plant diseases that produce severe economic losses worldwide. In the disease system, thrips not only damage plants through feeding but also transmit causative agents of epidemics. In addition, thrips are infected with tospoviruses in the course of virus transmission. Most studies on the effect of tospoviruses on vector thrips have focused on the *Tomato spotted wilt virus–Frankliniella occidentalis* system. Thus, we focused on another thrips-borne tospovirus, *Watermelon silver mottle virus* (WSMoV), to examine the effect of virus infection on its vector, *Thrips palmi*. In this study, the direct and indirect effects of WSMoV on the life history traits and feeding preference of *T. palmi* were examined. The survival rate and developmental time of the WSMoV-infected larval thrips did not differ significantly from those of the virus-free thrips. Comparing the developmental time of larval thrips fed on the healthy plants, thrips-damaged plants, and thrips-inoculated plants (the WSMoV-infected plants caused by thrips feeding), feeding on the thrips-damaged plants reduced the developmental time, and the WSMoV infection in host plants partially canceled the effect of thrips damage on the developmental time. In addition, no significant variations between the virus-free and WSMoV-infected adult thrips regarding longevity and fecundity were observed. These results implied that WSMoV did not directly affect the life history traits of *T. palmi*, but the WSMoV infection indirectly affected the development of *T. palmi* through the virus-infected plants. Furthermore, feeding preference tests indicated that *T. palmi* preferred feeding on either the thrips-damaged plants or the thrips-inoculated plants to the healthy plants. The effect of tospoviruses on the life history and feeding preference of vector thrips might vary among host plants, virus species, vector species, and environmental factors.

## Introduction

The majority (76%) of plant viruses is vector borne, and most of them are transmitted by hemipteran insects [1]. Insect-transmitted plant viruses cause numerous plant diseases that produce severe economic losses worldwide. Despite thrips-borne viruses account for only 2% of described plant viruses [1], they cause various crop disease epidemics of economic and social significance [2], [3], [4]. Thrips-borne *Tomato spotted wilt virus* (TSWV) is an infamous plant virus worldwide. TSWV can infect more than 900 plant species, and at least nine thrips species are reported to transmit the virus from plant to plant [3], [5]. TSWV is persistent and replicates within its vector thrips similarly to other tospoviruses [6], [7]; thus, thrips play two roles in the disease system: host and vector.

The successful transmission of insect-transmitted plant viruses primarily depends on the interactions between insects and viruses. Insects may carry plant viruses in their stylets, foreguts, or salivary glands, and inoculate viruses when they feed on healthy plants [8]. Many plant viruses (i.e. nonpersistently and semipersistently transmitted viruses) are only retained in the stylets or foreguts of their vector insects [9]. Other plant viruses (i.e. persistently transmitted viruses) infect insect tissues, including the salivary glands, and may replicate in vector insects [1]. Depending on the occurrence of virus multiplication in vector insects, the persistent transmission mode is further divided into two subcategories, persistent-circulative and persistent-propagative modes [1], [8]. Thrips-transmitted tospoviruses are regarded as persistent-propagatively transmitted viruses [7].

Numerous examples of animal and even plant viruses that affect the fitness of their vector insects have been presented (e.g., [10], [11], [12], [13], [14]). Thrips not only transmit tospoviruses but are also infected with the viruses [15], [16], [17], [18]; thus, tospoviruses likely affect the survival and fecundity of their vector thrips. However, no detrimental effects on the life cycle or cytopathological changes were observed in the vector thrips after TSWV infection [6], [19]. According to our literature review, few studies have been conducted on the direct effect of tospoviruses on their vector thrips.

In addition to the direct effect, plant viruses may indirectly affect vector insects through virus-infected host plants. The infection of tospoviruses likely affects various physiological properties of host plants and changes their nutritive value or secondary plant compounds [20], [21], [22]. Secondary plant compounds may repel insects or inhibit feeding and/or oviposition at the behavioral level and poison insects or decrease food quality at the physiological level. The indirect effect of tospoviruses on their vector thrips has been well studied, and the results vary from negative [13], [23], [24], to neutral [19], [25], and to positive [26], [27], [28], [29]. More studies have demonstrated that tospovirus-infected plants are beneficial to vector thrips. The majority of these studies focused on TSWV and its primary vector, *Frankliniella occidentalis*.

Most studies on the indirect effect of tospoviruses on vector thrips have focused on the TSWV – *F. occidentalis* system, but assuming that all tospoviruses affect their plant hosts and vector thrips similarly to the well-studied prototype TSWV is risky. We focused on another thrips-transmitted tospovirus, *Watermelon silver mottle virus* (WSMoV), to examine the direct and indirect effects of WSMoV on its vector, *Thrips palmi*. Tospoviruses are divided into two major groups, Asia and Americas, based on the phylogenetic analysis of nucleoprotein amino acid sequences [3]. WSMoV is a representative of the Asian group and is distantly related to TSWV, a representative of the American group. The only confirmed vector for WSMoV is *T. palmi* [30]. The results of this study are useful for improving our understanding of the effect of tospoviruses on vector thrips.

Previous studies on the effect of tospoviruses on vector thrips were performed on either the healthy or the virus-infected host plants. The virus could be inoculated to host plants by the viruliferous thrips, and the effect of the physiological and nutritional status of the virus-infected plants on the fitness of vector thrips might overwhelm the effect of virus infection in the thrips. It was difficult to discriminate between the direct and indirect effects of tospoviruses on vector thrips. We carefully designed our experiments and used bean seedlings as nonhosts for WSMoV, but suitable host plants for *T. palmi*. This study examined the direct and indirect effects of WSMoV on the life history traits of its vector, *T. palmi*. In addition, the feeding preference of *T. palmi* for the WSMoV-infected plants was also tested. The effects of WSMoV on the life history traits and feeding preference of *T. palmi* were compared with those of other tospovirus-thrips systems.

## Materials and Methods

### Ethics statement

The virus-infected samples were collected from a commercial farm (GPS coordinates: 23.21017, 120.169373) in Tainan, Taiwan. The owner agreed us to collect the samples. No specific permissions were required for the location.

### Insects, viruses, and plants

The *T. palmi* colony, derived from field-collected specimens on eggplants (*Solanum melongena*) on the experimental farm at National Taiwan University, was reared on bean (*Phaseolus coccineus*) seedlings in a growth chamber at 25°C, 70% relative humidity, and a photoperiod of L:D 16∶8 h. A bean seedling at the two-true leaves stage was enclosed with thrips in a 2-L beaker covered with a fine screen (150 mesh). The plant was replaced with a new seedling every 3 d. To obtain cohorts of first-instar larvae, the adult female thrips were allowed to lay eggs on cucumbers (*Cucumis sativus*), and pollen was provided to enhance oviposition. To obtain viruliferous thrips, the newly hatched larvae (<6 h) were allowed to feed on the WSMoV-infected leaves of watermelons for 24 h to acquire the virus. More than 90% of these larvae were positive for WSMoV as detected by a reverse-transcription polymerase chain reaction (RT-PCR, described in the subsequent section) analysis. The control group (virus-free thrips) included the newly hatched larvae that were allowed to feed on the healthy watermelon leaves for 24 h.

The WSMoV Tainan isolate was derived from field-collected leaves of watermelons (*Citrullus lanatus*) on a commercial farm in Tainan, Taiwan. The virus culture was maintained in the watermelon seedlings by thrips-mediated passage to prevent the loss of thrips transmissibility. The adult female thrips were allowed to lay eggs on the WSMoV-infected seedlings, and the subsequent generation of adult thrips was used to inoculate the healthy watermelon seedlings. The watermelon seedlings with apparent symptoms were used as virus sources approximately 2 to 3 wk postinoculation. The virus source plants and healthy plants were contained in separate growth chambers under the same aforementioned environmental conditions.

All of the plants (bean and watermelon) used in the experiments were grown from seeds under the same environmental conditions. To obtain the WSMoV-infected plants caused by the feeding of viruliferous thrips (subsequently referred to as “thrips-inoculated plants”), the healthy watermelon seedlings at the two-true leaves stage were caged with a population of viruliferous thrips in a 2-L beaker for 2 wk. To obtain plants with thrips damage but without WSMoV infection (subsequently referred to as “thrips-damaged plants”), the healthy watermelon seedlings at the same stage were enclosed with a population of virus-free thrips in a 2-L beaker for 2 wk. After 2 wk, all visible thrips were inspected and removed using a fine brush.

### Virus detection

The total RNA was extracted from single thrips or plant tissues by using TRIzol Reagent (Invitrogen, Carlsbad, CA, USA) according to the manufacturer’s instructions. An RT-PCR analysis was conducted using the OneStep RT-PCR kit (Qiagen, Valencia, CA, USA), template RNA, and WSMoV specific primer pair, WSMoV848 (5′-ATCACCATAATCATCCACAG-3′) and WSMoVR (5′-GAGAGAGCAATCGAGGC-3′), modified from Uga and Tsuda [31]. The reverse transcription was performed at 50°C for 30 min, followed by a PCR activation step at 95°C for 15 min, an amplification of 40 cycles at 94°C for 30 s, 50°C for 30 s, and 72°C for 1 min, and a final extension step at 72°C for 10 min. A PCR product of 851 bp was predicted for the WSMoV-infected materials.

### Direct effect of WSMoV on the survival and development of thrips

To examine the direct effect of WSMoV on *T. palmi*, the survival rate and developmental time of the virus-free and viruliferous larval thrips were measured on the bean leaves. The bean leaf was used because it is a good food source for *T. palmi* and is not susceptible to WSMoV. The viruliferous thrips could not transmit the virus to the bean leaf to change the nutritive value of the leaf. The virus-free or viruliferous larva was individually reared on a piece of bean leaf (1 cm^2^) in a Petri dish (3.5 cm in diameter). The Petri dishes were sealed with parafilm to prevent the larvae from escaping and were incubated at 25°C, 70% relative humidity, and a photoperiod of L:D 16∶8 h. The leaflets were replaced daily. The survival and developmental stage of the larvae were recorded every 12 h until the larvae reached the prepupal stage. The functions describing the survival rates from the first-instar to the prepupal stage were estimated using the Kaplan-Meier method and subjected to survival analysis [32]. The survival rates and developmental times of the virus-free and viruliferous larvae were analyzed using the log-rank test and the negative binomial distribution test, respectively.

### Indirect effect of WSMoV on the survival and development of thrips

To examine the effect of WSMoV on *T. palmi* through host plants, the survival rate and developmental time of the virus-free and viruliferous larval thrips reared on the healthy, thrips-damaged, and thrips-inoculated watermelon leaves were measured. The virus-free and viruliferous larvae were individually reared on a piece of the healthy, thrips-damaged, or thrips-inoculated watermelon leaves (1 cm^2^) in a Petri dish under the same aforementioned environmental conditions. The leaflets were replaced daily. The survival and developmental stage of the larvae were recorded every 12 h until the larvae reached the prepupal stage. The functions describing the survival rates from the first-instar to the prepupal stage were estimated using the Kaplan-Meier method and subjected to survival analysis. The survival rates and developmental times of the virus-free and viruliferous larvae were analyzed using the log-rank test and the negative binomial distribution test, respectively.

We further measured the head capsule width of the pupae derived from these virus-free larvae as an indicator to examine the indirect effect of the WSMoV infection on the body size of thrips. The head capsule widths of the pupae were analyzed using the negative binomial distribution test.

### Direct effect of WSMoV on the longevity and fecundity of thrips

The direct effect of WSMoV on the longevity and fecundity of *T. palmi* was examined with the virus-free and viruliferous adult thrips reared on the bean leaves. The virus-free and viruliferous larvae were transferred to bean seedlings until they emerged as adults. The adult thrips were sexed and individually reared on a piece of bean leaf (1 cm^2^) in a Petri dish under the same aforementioned environmental conditions. The leaflets were replaced daily. The number of eggs that a single female produced was recorded daily until it died. The leaflets were stained with lactophenol-acid fuchsin solution to facilitate egg counting under a stereomicroscope [33]. The life-long egg production of the virus-free and viruliferous thrips was analyzed using the Student *t* test. The longevity of the virus-free and viruliferous adult males and females was also recorded and analyzed using the Student *t* test.

### Feeding preference of adult thrips for the WSMoV-infected plants

The effect of the WSMoV-infected plants on the feeding preference of *T. palmi* was examined with virus-free adult thrips. Two test plants (watermelon seedlings) were placed in a transparent acryl cage (30×30×30 cm^3^) and separated by 16 cm for choice tests. The test plants were a healthy plant versus a thrips-damaged plant, a healthy plant versus a thrips-inoculated plant, and a thrips-damaged plant versus a thrips-inoculated plant. Ten adult thrips were caged in a 1.5-ml microcentrifuge tube and then released at a spot between two test plants at equal distances (8 cm) from each plant. The movement of the thrips was recorded by counting the number of thrips that remained on each plant at 2 h, 4 h, 8 h, 12 h, and 24 h after the release of the thrips. The experiments were performed under the same aforementioned environmental conditions. Each experiment was repeated three times. The male and female thrips were examined separately to determine the feeding preference between the sexes. The results of the choice tests were analyzed using a sign test.

## Results

### Direct effect of WSMoV on the survival and development of thrips

**Figure 1:**
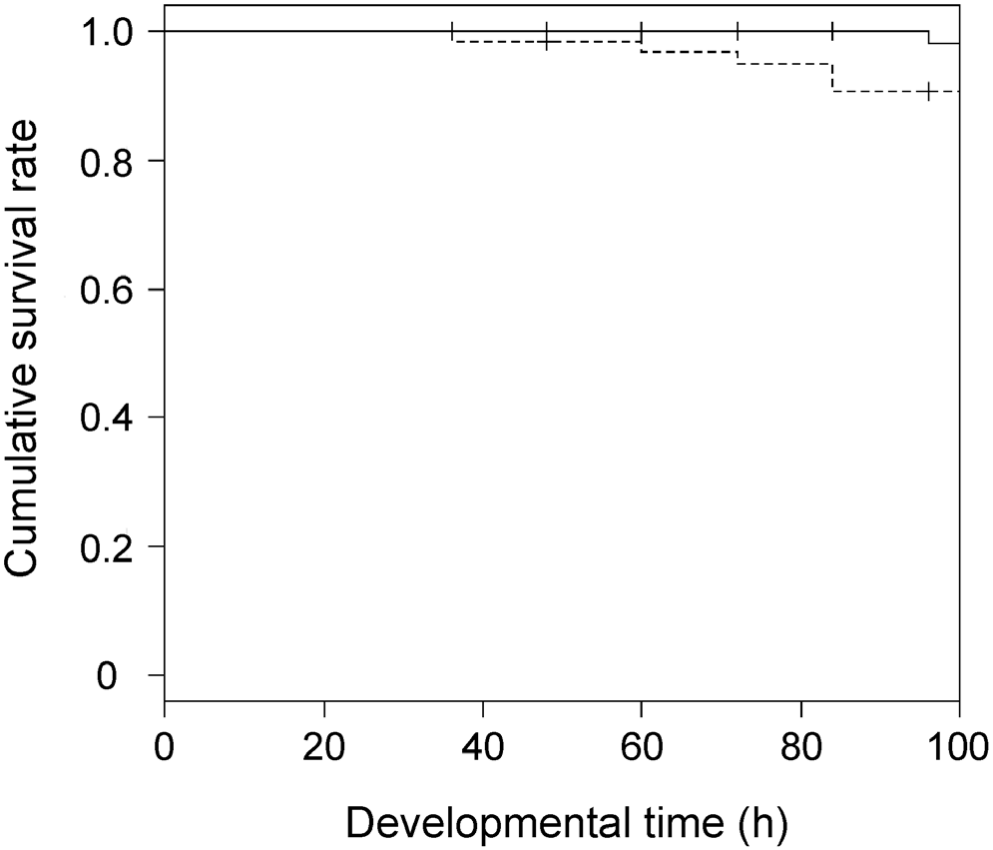
Cumulative survival rate of *Thrips palmi* larvae (Kaplan-Meier). The virus-free larvae were reared on the bean leaves (n = 65, solid line). The viruliferous (infected with *Watermelon silver mottle virus*) larvae were reared on the bean leaves (n = 70, dashed line).

The direct effect of WSMoV on *T. palmi* was assessed based on the survival rates and developmental times of the virus-free and viruliferous larvae fed on the bean leaves (nonhost for WSMoV). The cumulative survival rates of the virus-free and viruliferous larvae are shown in Fig. 1 and subjected to survival analysis. No significant variation between the survival rates of the virus-free and viruliferous larvae was observed (Table 1; log-rank test, *P* = 0.09). The developmental times from the first-instar to the prepupal stage of the virus-free and viruliferous larvae fed on the bean leaves were 119.4±31.6 h (mean ± SD; n = 38) and 122.9±18.4 h (n = 37), respectively. No significant variation between the developmental times of the virus-free and viruliferous larvae was observed (Table 1; negative binomial distribution test, *P* = 0.73). The results implied that WSMoV did not directly affect the survival and development of *T. palmi.*

**Table 1:** Direct effect of *Watermelon silver mottle virus* on the survival rate and developmental time from the first-instar to the prepupal stage of *Thrips palmi* on the bean leaves.

Life history trait	Test statistic	*P* value
Survival rate	2.81^a^	0.09
Developmental time	−339.09^b^	0.73

a The log-rank test was used for planned comparison.

b The negative binomial distribution was used for planned comparison.

### Indirect effect of WSMoV on the survival and development of thrips

**Figure 2:**
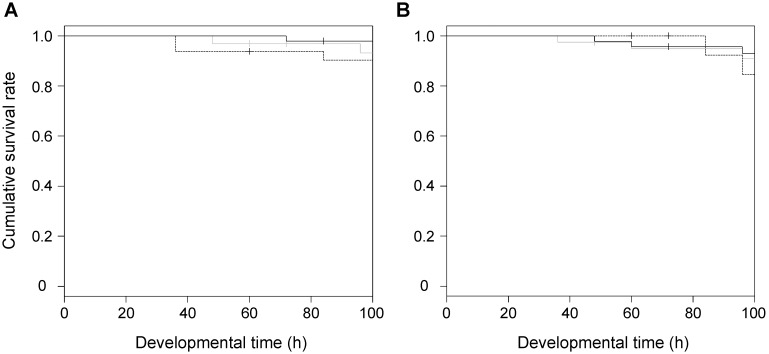
Cumulative survival rate of *Thrips palmi* larvae (Kaplan-Meier). (**A**) The virus-free larvae were reared on the healthy watermelon leaves (n = 47, solid line), on the thrips-damaged leaves (n = 32, dashed line), and on the thrips-inoculated leaves (n = 32, grey line). (**B**) The viruliferous (infected with *Watermelon silver mottle virus*) larvae were reared on the healthy watermelon leaves (n = 46, solid line), on the thrips-damaged leaves (n = 26, dashed line), and on the thrips-inoculated leaves (n = 40, grey line).

The indirect effect of WSMoV on *T. palmi* was assessed based on the survival rates and developmental times of the virus-free and viruliferous larvae fed on the healthy, thrips-damaged, and thrips-inoculated watermelon leaves. The cumulative survival rates of the virus-free and viruliferous larvae are shown in Fig. 2 and subjected to survival analysis. The survival rates of the virus-free larvae did not differ significantly from the larvae fed on the healthy, thrips-damaged, or thrips-inoculated leaves (Table 2; log-rank test, *P* = 0.31). Similarly, the survival rates of the viruliferous larvae did not differ significantly among these three treatments (Table 2; log-rank test, *P* = 0.17). The results suggested that thrips damage and the WSMoV infection of host plants did not affect the survival of *T. palmi.*

**Table 2:** Indirect effect of *Watermelon silver mottle virus* (WSMoV) on the survival rate from the first-instar to the prepupal stage of *Thrips palmi* on the watermelon leaves.

Thrips status	Test statistic	*P* value
Virus-free	4.60^a^	0.31
Viruliferous (WSMoV)	3.50^a^	0.17

a The log-rank test was used for planned comparison.

**Figure 3:**
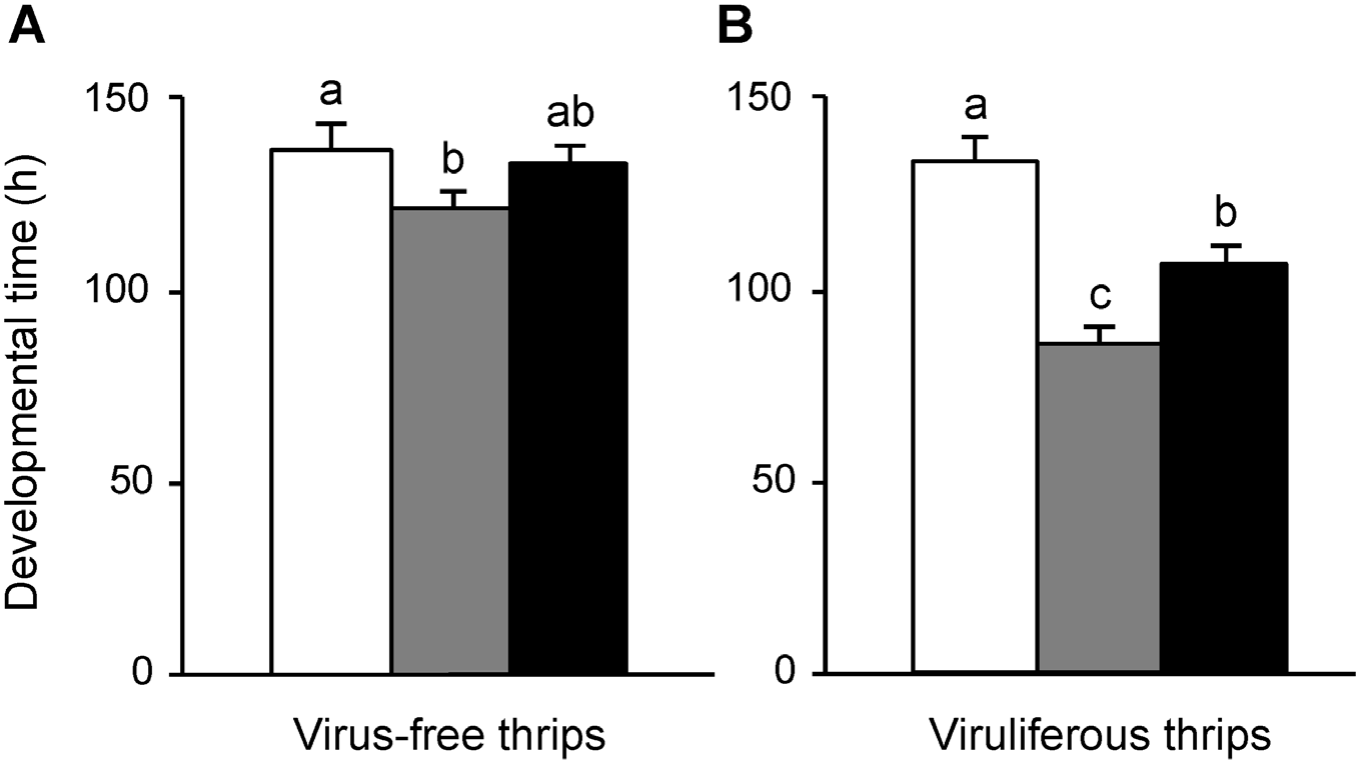
Developmental time from the first-instar to the prepupal stage of *Thrips palmi* reared on the watermelon leaves. (**A**) The virus-free larvae were reared on the healthy leaves (n = 32, white column), on the thrips-damaged leaves (n = 20, grey column), and on the thrips-inoculated leaves (n = 16, black column). (**B**) The viruliferous (infected with *Watermelon silver mottle virus*) larvae were reared on the healthy leaves (n = 28, white column), on the thrips-damaged leaves (n = 15, grey column), and on the thrips-inoculated leaves (n = 28, black column). Different letters indicate statistical differences between means at *P*<0.05, analyzed using the negative binomial distribution test. Vertical bars indicate standard error.

The developmental times of the virus-free and viruliferous larvae fed on the watermelon leaves of the three treatments are shown in Fig. 3. The developmental time of the virus-free larvae was significantly longer when they were fed on the healthy leaves than when they were fed on the thrips-damaged leaves (Table 3; negative binomial distribution test, *P*<0.05). The developmental times of the virus-free larvae did not differ significantly from the larvae fed on the healthy leaves or the thrips-inoculated leaves (Table 3; negative binomial distribution test, *P* = 0.53), nor from the larvae fed on the thrips-damaged leaves or the thrips-inoculated leaves (Table 3; negative binomial distribution test, *P* = 0.52). The variations of the developmental times among these treatments became more evident when *T. palmi* were infected with WSMoV. The developmental time of the viruliferous larvae was significantly longer when they were fed on the healthy leaves, compared with when they were fed on the thrips-damaged leaves (Table 3; negative binomial distribution test, *P*<0.05), and when they were fed on the thrips-inoculated leaves (Table 3; negative binomial distribution test, *P*<0.01). The developmental time of the viruliferous larvae was significantly longer when they were fed on the thrips-inoculated leaves than when they were fed on the thrips-damaged leaves (Table 3; negative binomial distribution test, *P*<0.01). The results suggested that feeding on the thrips-damaged leaves reduced the developmental time of *T. palmi*, and the WSMoV infection in host plants partially canceled the effect of thrips damage on the developmental time of *T. palmi*.

**Table 3:** Indirect effect of *Watermelon silver mottle virus* (WSMoV) on the developmental time from the first-instar to the prepupal stage of *Thrips palmi* on the watermelon leaves.

Comparison	Virus-free		Viruliferous (WSMoV)	
	Test statistic^a^	*P*	Test statistic^a^	*P*
Healthy vs. thrips-damaged	−235.10	<0.05	−203.92	<0.05
Healthy vs. thrips-inoculated	−220.80	0.53	−266.85	<0.01
Thrips-damaged vs. thrips-inoculated	−163.44	0.52	−193.58	<0.01

a The negative binomial distribution was used for planned comparison.

We further measured the size of the pupae derived from the larvae. The head capsule widths of the virus-free pupae reared on the healthy, thrips-damaged, and thrips-inoculated leaves were 1.38±0.15 mm (mean ± SD; n = 29), 1.28±0.14 mm (n = 31), and 1.32±0.14 mm (n = 31), respectively. However, no significant variation in the head capsule widths of the pupae among these three treatments was observed (Table 4; negative binomial distribution test, *P*>0.05). The results implied that thrips damage and the WSMoV infection in host plants did not affect the body size of *T. palmi.*

**Table 4:** Indirect effect of *Watermelon silver mottle virus* on the head capsule width of the virus-free pupae of *Thrips palmi* on the watermelon leaves.

Comparison	Test statistic^a^	*P* value
Healthy vs. thrips-damaged	−67.78	0.76
Healthy vs. thrips-inoculated	−68.15	0.86
Thrips-damaged vs. thrips-inoculated	−69.50	0.90

a The negative binomial distribution was used for planned comparison.

### Direct effect of WSMoV on the longevity and fecundity of thrips

The direct effect of WSMoV on *T. palmi* was assessed based on the longevity and fecundity of the virus-free and viruliferous adult thrips fed on the bean leaves. The longevity of the virus-free and viruliferous male thrips was 16.8±1.0 d (mean ± SD; n = 31) and 17.1±1.4 d (n = 29), respectively. The longevity of the virus-free and viruliferous female thrips was 18.0±2.0 d (n = 29) and 18.0±1.4 d (n = 27), respectively. For both sexes, no significant variation between the longevity of the virus-free and viruliferous thrips was observed (Table 5; Student *t* test, *P*>0.05). The number of eggs that the female thrips produced was 59.4±7.6 (mean ± SD; n = 29) for the virus-free thrips and 58.2±5.4 (n = 27) for the viruliferous thrips. No significant variation between the life-long egg production of the virus-free and viruliferous thrips was observed (Table 5; Student *t* test, *P* = 0.49). The results suggested that WSMoV did not directly affect the longevity and fecundity of *T. palmi.*

**Table 5:** Direct effect of *Watermelon silver mottle virus* on the longevity and fecundity of the virus-free adults of *Thrips palmi* on the bean leaves.

Life history trait	Test statistic	*P* value
Male longevity	−0.74^a^	0.46
Female longevity	−0.08^a^	0.94
Female fecundity	0.70^a^	0.49

a Student *t* test.

### Feeding preference of adult thrips for the WSMoV-infected plants

**Figure 4:**
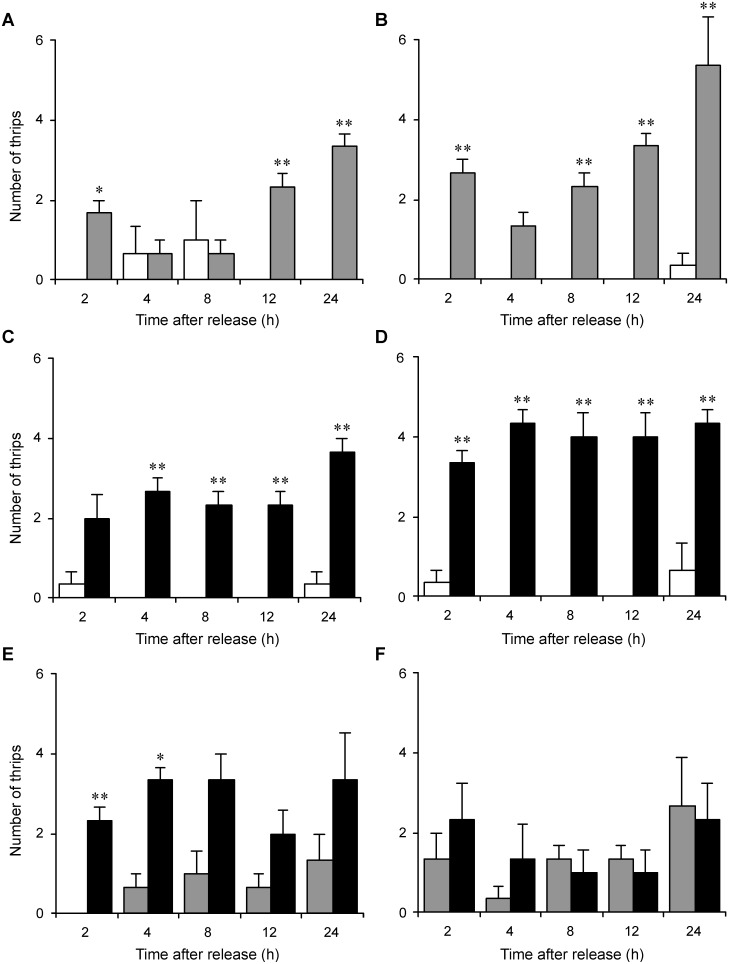
Feeding preference of adults of *Thrips palmi* for the watermelon seedlings. (**A**) Males and (**B**) females that fed on the healthy seedlings (white columns) and the thrips-damaged seedlings (grey columns). (**C**) Males and (**D**) females that fed on the healthy seedlings (white columns) and the thrips-inoculated seedlings (black columns). (**E**) Males and (**F**) females that fed on the thrips-damaged seedlings (grey columns) and the thrips-inoculated seedlings (black columns). Ten males or females were released between the two seedlings at the beginning of the preference experiment. The experiment was repeated three times. * and ** indicate statistical differences (*P*<0.05 and *P*<0.01, respectively) analyzed using a sign test. Vertical bars indicate standard error.

The effect of the WSMoV infection in plants on the feeding preference of *T. palmi* was examined with the virus-free adult thrips. The feeding preference was examined using choice tests between two watermelon seedlings. More male thrips fed on the thrips-damaged plants than on the healthy plants 2 h, 12 h, and 24 h after the release of the test thrips (Fig. 4A). Similarly, more female thrips fed on the thrips-damaged plants than on the healthy plants 2 h, 8 h, 12 h, and 24 h after the release of the test thrips (Fig. 4B). More male thrips fed on the thrips-inoculated plants than on the healthy plants 4 h, 8 h, 12 h, and 24 h after the release of the test thrips (Fig. 4C). Similarly, more female thrips fed on the thrips-inoculated plants than on the healthy plants 2 h, 4 h, 8 h, 12 h, and 24 h after the release of the test thrips (Fig. 4D). The numbers of male and female thrips that fed on the thrips-damaged plants and on the thrips-inoculated plants did not significantly differ 8 h, 12 h, and 24 h after the release of the test thrips (Figs. 4E and 4F). The results indicated that *T. palmi* preferred feeding on either the thrips-damaged or thrips-inoculated plants than on the healthy plants.

## Discussion

Few studies have analyzed the direct effect of tospoviruses on vector thrips. We carefully designed our experiments and used bean seedlings as host plants for *T. palmi* to examine the direct effect of WSMoV on the survival, development, longevity, and fecundity of *T. palmi*. The indirect effect of the WSMoV infection on the survival, development, and feeding preference of *T. palmi* was examined using the healthy, thrips-damaged, and thrips-inoculated watermelon plants. Our results suggested that WSMoV did not directly affect the survival, development, longevity, and fecundity of *T. palmi.* An indirect effect of the WSMoV infection on the development of *T. palmi* through the host plant was observed. The direct and indirect effects of WSMoV on the life history traits of *T. palmi* were compared with those of other tospovirus-thrips systems.

Most plant-pathogenic viruses are benign to their insect vectors [1], [8]. Our results suggested that WSMoV did not directly affect the survival, development, longevity, and fecundity of *T. palmi* reared on the healthy plants (Tables 1 and 5). WSMoV may not heavily infect the tissues of *T. palmi* and not cause cytopathological effects in its vector thrips so as the TSWV infection in *F. occidentalis* [6], [19]. However, no studies have been conducted on the issue for the WSMoV – *T. palmi* system. Belliure et al. [27] also reported no direct effect of TSWV on the survival and development of *F. occidentalis* larvae raised on healthy or thrips-inoculated plants. However, TSWV positively affected the survival and development of *F. occidentalis* larvae raised on mechanically inoculated plants [27]. As the comparison was made on the TSWV-infected leaves, this difference might be attributed to an interaction between the direct and indirect effects of the virus. Researchers have attempted to analyze the direct effect of TSWV on *F. occidentalis*, but most studies could not exclude the interaction between TSWV and host plants. For example, the virus-free and viruliferous thrips were fed on the healthy and virus-infected plants, respectively; therefore the test thrips were fed on host plants with different infection statuses. The comparison was not based on an equitable basis.

Most studies on the tospovirus-vector interaction have focused on the indirect effect of virus infection on vector thrips, particularly on the TSWV – *F. occidentalis* system. The egg stage and larval stage of *F. occidentalis* were shorter on the TSWV-infected plants than on the healthy plants, but the developmental time of the prepupal stage and pupal stage did not change [28]. This variation may be due to the nonfeeding nature of the prepupa and pupa of thrips. Belliure et al. [27] further discovered that *F. occidentalis* larvae raised on the thrips-damaged plants exhibited a lower survival rate and longer developmental time than those raised on the thrips-inoculated plants or the healthy plants. The results suggested that the quality of the host plants decreases after the thrips’ feeding and then it adversely affects the survival and development of *F. occidentalis.* The TSWV infection in the thrips-inoculated plants offsets the negative effects of thrips damage. The feeding damage by *F. occidentalis* triggers the jasmonate (JA)-regulated defense of host plants, which adversely affects the population of *F. occidentalis* [34]. The TSWV infection induces the salicylic acid-regulated plant defense that antagonizes the thrips-induced JA-regulated defense [26], [35]. The TSWV infection suppresses the thrips-induced JA-regulated defense, so the negative effects caused by the thrips’ feeding are offset. This offsetting may explain the aforementioned positive indirect effect of TSWV on *F. occidentalis*.

We discovered that the WSMoV infection in host plants did not affect the survival of *T. palmi* (Fig. 2), but the larval development of *T. palmi* was affected when they were reared on the thrips-damaged plants or the thrips-inoculated plants (Fig. 3). The indirect effect of the WSMoV infection on its vector thrips differed from that of the TSWV infection on its vector thrips. The larval development of *F. occidentalis* reared on the thrips-damaged plants was slower than that of those reared on the healthy plants or the thrips-inoculated plants, and no thrips reared on the thrips-damaged plants reached the prepupal stage [27]. Our results indicated that feeding on the thrips-damaged plants reduces the developmental time of *T. palmi*, and the WSMoV infection in host plants partially cancels the effect of thrips damage on the developmental time of *T. palmi* (Fig. 3). We propose two hypotheses to explain why *T. palmi* grows faster on the thrips-damaged plants than on the healthy plants. A hypothesis is that *T. palmi* pupates earlier because the thrips-damaged plants are less favorable for *T. palmi* than the healthy plants or the thrips-inoculated plants. We further examined the head capsule widths of pupae of *T. palmi* reared on the healthy, thrips-inoculated, and thrips-damaged plants. The results showed that thrips damage and the WSMoV infection in host plants did not affect the body size of *T. palmi*, which suggests that *T. palmi* grows faster on the thrips-damaged plants without compromising its body size. Therefore, this hypothesis is refuted. Another hypothesis is that the thrips-damaged plants are covered with conspecific pheromones from previous treatments, and the pheromone expedites the development of the test thrips. *Frankliniella occidentalis, F. intonsa*, and *F. schultzei* were reported to secrete pheromones to attract conspecific male and female thrips [36], [37], [38]; however, there is no study on the effect of pheromone on thrips development. Stumpf and Kennedy [24], [29] and Shrestha et al. [13] reported both direct and indirect effects of TSWV on the fitness of *F. occidentalis* and *F. fusca,* and they also provided evidences that those inconsistent results from various tospovirus-vector systems might derive from different host plants, virus isolates, vector species, and environmental factors.

Plants infected with viral pathogens are more attractive to hemipteran insects and thrips [28], [39], [40], [41]. The TSWV-infected plants attract more *F. occidentalis* than the healthy plants do [28], [42]. In addition, male thrips infected with TSWV fed more frequently compared with healthy males [43]. If vector insects are more attracted to the virus-infected plants and viruliferous vectors are more active to feed, the behavior promotes the spread of viral diseases. In this study, *T. palmi* preferred feeding on the thrips-damaged plants and thrips-inoculated plants than on the healthy plants (Fig. 4). The preference for the thrips-damaged plants and thrips-inoculated plants did not differ between the male and female thrips. The results suggested that previous thrips feeding renders the host plants more attractive to *T. palmi* regardless of the infection status of WSMoV in the plants. This finding is consistent with our inference that conspecific pheromones from previous treatments might attract the test thrips.

The control of insect-transmitted plant viral diseases not only requires knowledge of virus-plant interactions but the life history traits of vector insects must also be considered. These life history traits (e.g., survival rate, developmental time, longevity, fecundity) are affected by the direct and indirect effects of plant viruses. Both the direct and indirect effects of tospoviruses can potentially affect the population of the viruliferous thrips that feed on the virus-infected host plants, and thereby, influence the spread of tospoviruses from these plants. The effects of tospoviruses on the life history and feeding preference of vector thrips might vary among host plants, virus species, vector species, and environmental factors. Understanding the ecological interactions between tospoviruses and their vector thrips is critical to protect crops from thrips-transmitted plant diseases.
